# Structural Inequities and Chronic Disease in Fresno County: A Narrative Review and Multi-sectoral Framework

**DOI:** 10.7759/cureus.112418

**Published:** 2026-07-10

**Authors:** Adersin Vartanyan, Pauline Tran, Karen Bontekoe, Alvaro Pinto

**Affiliations:** 1 Department of Biomedical Education, California Health Sciences University College of Osteopathic Medicine, Clovis, USA; 2 Health Sciences Library, California Health Sciences University College of Osteopathic Medicine, Clovis, USA

**Keywords:** chronic disease epidemiology, environmental justice, fresno county, health public, health status disparities, social determinants of health

## Abstract

Despite California's favorable statewide health profile, significant intra-state disparities exist. Fresno County, located in the Central Valley, consistently ranks among the state's worst-performing counties for health outcomes, presenting a critical case study in health inequity. This study analyzes the prevalence of chronic diseases in Fresno County and investigates the underlying factors contributing to this public health crisis. A comparative analysis was conducted using public health data from 2018 to 2021 to assess the prevalence of major chronic diseases, including obesity, hypertension, diabetes, asthma, coronary heart disease (CHD), and stroke. The prevalence rates in Fresno County were compared with those in California, considering social determinants of health impacting Fresno residents. Fresno County exhibits significantly higher prevalence and mortality rates for most major chronic diseases when compared to regional and statewide averages. These adverse health outcomes are strongly correlated with a complex web of interconnected factors, including pervasive poverty, low educational attainment, critical shortages of healthcare providers, severe air and pesticide pollution, and widespread food insecurity. These determinants disproportionately impact the county's racial and ethnic minority populations, exacerbating health disparities. The findings establish that Fresno's chronic disease burden is a direct consequence of structural inequities. We argue that an actionable framework targeting socioeconomic determinants, environmental justice, and healthcare system reform is imperative to address these structural inequities.

## Introduction and background

Health landscape: California and Fresno County

California exhibits a favorable health profile compared to the national average. In 2021, California recorded the 45th-highest age-adjusted death rate, indicating a lower overall mortality burden than most other states [1, 2]. This improved mortality picture is further supported by the state's heart disease death rate, which was 147.8 per 100,000 residents in 2021, nearly 15% lower than the national average of 173.8 [2, 3]. Additionally, California ranks as the 4th-highest state for life expectancy at birth in the U.S., with newborns expected to live 79.0 years, a full 2.0 years longer than the national average [2, 4]. These statistics establish California as a state with generally robust health indicators.

However, certain regions in California, such as Fresno County and the overarching Central Valley, have demonstrated alarming disparities compared to California's overall health profile. This situation is not merely an isolated issue of poor health outcomes in one county; rather, it indicates that Fresno County's health statistics actively diminish the regional and statewide averages. This phenomenon highlights a significant intra-state equity challenge. California’s overall health success is not equitably shared across its diverse communities.

Central Valley's health disparities

The Central Valley, a region renowned for its extensive agricultural production, simultaneously grapples with profound socioeconomic and health disparities. This area is home to nearly 4 million people, comprising over 70 ethnicities and 105 languages, signifying a remarkably diverse population [5]. However, this rich cultural tapestry coexists with widespread hardships that impact public health. For example, food insecurity is prevalent, affecting up to 20% of the population in many counties. Educational attainment is notably low, with three Central Valley metropolitan areas ranking among the top 10 U.S. metropolitan areas for the lowest average education in 2013. Poverty levels are also high, with seven counties falling below the national poverty threshold, with Tulare County registering a staggering 23.8% of its population below this level [5].

Aside from socioeconomic challenges, the Central Valley is burdened by severe environmental pollution. Four cities within the region were among the top 5 most polluted in the American Lung Association's 2017 "State of the Air" report [5]. A severe physician shortage exacerbates these health challenges. The San Joaquin Valley, a sub-region of the Central Valley, averages significantly fewer primary care doctors (48 per 100,000 residents) and specialists (80 per 100,000) compared to San Francisco (86 and 175, respectively) [5]. These interconnected factors ultimately contribute to poorer health outcomes for the Valley's vulnerable populations [6]. The unique combination of agricultural wealth and widespread poverty in the Central Valley creates a stark paradox: the land that yields a substantial portion of the nation's food supply fails to adequately nourish its residents or provide them with essential health infrastructure. These findings underscore entrenched structural inequities, where economic models prioritize production over local well-being, perpetuating cycles of deprivation despite regional prosperity [7].

Fresno County: a critical case study

Fresno County is a prime illustration of the Central Valley's pervasive challenges, consistently ranking among the worst health outcomes in California. It is specifically identified for its disproportionately high burden of chronic diseases. The county's population demographics reveal a higher proportion of Latino residents (52% compared to California's 39%) and nearly double the child poverty rate (36.5% versus 20.8% statewide) [8]. These demographic and socioeconomic characteristics underscore the heightened vulnerability of its residents.

The 2022 Community Medical Center's Community Health Needs Assessment has identified stroke, heart attack, asthma, arthritis, obesity, diabetes, and high blood pressure as the most prevalent chronic diseases within Fresno County [8]. This report meticulously analyzed these specific conditions to illuminate the extent of the county's health burden. Fresno County acts as a microcosm of the broader Central Valley's health crisis, making it an ideal focus for understanding how systemic inequities manifest through health statistics. Despite California's overall health standing, the county's persistently poor ranking indicates that statewide health policies and resource allocations may not adequately address the unique, compound challenges faced by regions like Fresno. This suggests a need for a more nuanced approach to public health policy. Fresno is not merely an outlier but a crucial indicator of health equity challenges within a seemingly progressive state. Studying Fresno provides valuable lessons for developing targeted interventions that can be scaled or adapted for other similar regions, moving beyond a "one-size-fits-all" approach to public health policy. As the most populous county and the economic and demographic anchor of the San Joaquin Valley, Fresno serves as an essential case study for understanding the region's health dynamics.

Beyond documenting these disparities, this paper concludes by proposing an integrated, multi-sectoral framework for action, aligning with a growing consensus on the need to address the root causes of health inequity.

## Review

Comparative analysis of chronic disease burden

Overall Chronic Disease Prevalence Rates: Fresno County, San Joaquin Valley, and California

Central California includes the Sacramento Valley in the north and the San Joaquin Valley in the south. The San Joaquin Valley (SJV) is a prominent agricultural region, with Fresno County's largest city, Fresno, situated at its center and serving as a key hub for the region's agriculture, culture, and education.

**Figure 1:**
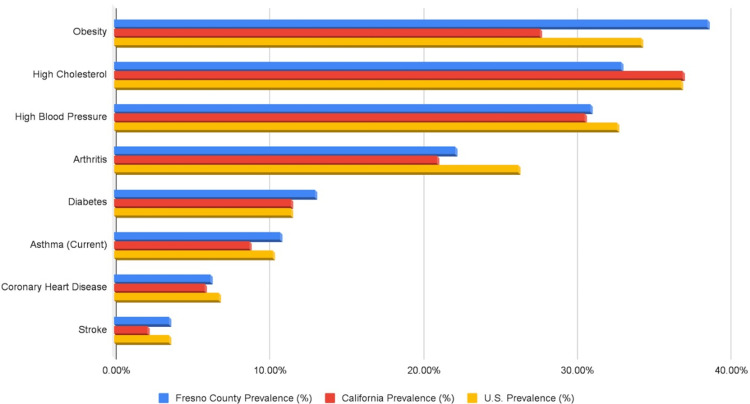
Average Prevalence Rates of Chronic Diseases Average prevalence rates of major chronic diseases among adults (≥18 years) in Fresno County (CDC PLACES, 2022) [8], California (BRFSS, 2023) [9], and the U.S. (BRFSS, 2023) [10]. Image created by the authors using Google Sheets (Alphabet Inc, Mountain View, USA).

Fresno County generally exhibits higher prevalence rates for chronic diseases or conditions when compared to both the SJV region and statewide California averages (Figure 1) [7]. The most prevalent chronic diseases in Fresno County include obesity, high blood pressure, high cholesterol, and arthritis. A notable exception to Fresno's overall chronic condition of prevalence is high cholesterol, whose rates are not significantly higher than the SJV region or statewide averages.

Figure 1 compares average prevalence rates for major chronic diseases among adults (≥18 years) in Fresno County, California, and the U.S. from 2022 to 2023. Despite California's lower prevalence rate of chronic diseases when compared to the national level, Fresno County exhibits higher rates in most conditions, highlighting significant localized health disparities [8-12].

Disease-specific prevalence and key trends

Obesity

Fresno County: According to CDC PLACES 2022 [10], the estimated prevalence of obesity among adults aged 18 years and older in Fresno was 38.6 (95% CI: 33.3%-44.2%). This compares to 27.7% in California and 34.3% in the U.S [11,12]. Obesity consistently ranks as a predominant chronic condition in Fresno County, with minimal variation between 2018 and 2021 [7]. An analysis of adult obesity prevalence in Fresno County from 2018 to 2022 indicates that rates have fluctuated but remained elevated across Hispanic, White Non-Hispanic, and Black Non-Hispanic populations. While the Multiple-Race Non-Hispanic group exhibited the highest rates, a declining trend has been observed since 2019. Conversely, the Asian population, historically showing the lowest rates, experienced a significant increase in 2022.

U.S. National Data: The prevalence of obesity among U.S. adults aged 40-59 is lower compared to other age groups and is lower in adults with a bachelor's degree or more education [13]. The prevalence of severe obesity, a more extreme form, increased from 7.7% to 9.7% from 2013-2014 to 2021-2023. 

The persistently high rates of obesity stem from an entrenched lifestyle and socioeconomic factors despite current public health interventions [14]. In contrast to historically lower rates of obesity, the observed increase in obesity rates among Asians in Fresno in 2022 warrants further investigation into community-level factors, such as dietary shifts, acculturation impacts, or changes in access to healthy food options and physical activity opportunities. Better insight can lead to the formation of culturally informed strategies to improve public health outcomes.

High Cholesterol (Hypercholesterolemia)

Fresno County: According to CDC PLACES 2022 [10], the estimated prevalence of high cholesterol among adults aged 18 years and older in Fresno was 32.7 (95% CI: 29.2%-37.4%). This compares to 37.0% in California and 36.9% in the U.S. Between 2017 and 2019, the prevalence rates for hypertension and hypercholesterolemia in Fresno County were similar [7]. However, in 2021, high cholesterol prevalence surpassed hypertension's, showing slight fluctuations and peaking in that year. Despite these internal trends, Fresno County's average prevalence rates for high cholesterol (2018-2021) are not higher than those in the San Joaquin Valley region or statewide California averages, positioning it as an exception among the chronic diseases examined. 

U.S. National Data: Between 2017 and 2020, 10% of U.S. adults aged 20 or older had total cholesterol levels exceeding 240 mg/dL [15]. More recently, the age-adjusted prevalence of high total cholesterol in adults was 11.3% from August 2021 to August 2023 [15]. It is important to note that high cholesterol frequently presents without symptoms, making regular blood tests crucial for its detection and management. 

Fresno County presents a notable divergence in its hypercholesterolemia disease profile. While the prevalence of stroke and coronary heart disease (CHD) is significantly higher than in California, the reported rates of hypercholesterolemia, a primary cause of these conditions [16,17], do not appear to be similarly elevated. This discrepancy suggests that the prevalence of high cholesterol may be underrepresented due to insufficient screening. Data supports this hypothesis. At 81.1%, Fresno County's cholesterol screening rate falls into the lowest quartile for counties in both California (<82.7%) and the nation (<82.0%) [18]. Furthermore, within the county's 57 zip codes, screening rates vary from a low of 48.5% to a high of 90.7%, with a median of 79.6%. Because high blood cholesterol is asymptomatic, these lower screening rates likely lead to a sizable, undiagnosed population, thus increasing the incidence of its long-term complications.

High Blood Pressure (Hypertension)

Fresno County: According to CDC PLACES 2022 [10], the estimated prevalence of high blood pressure (HBP) among adults aged 18 years and older in Fresno was 30.8 (95% CI: 27.5%-34.9%), compared to 30.6% in California and 32.7% in the U.S. [7].

Hypertension has been identified as another prevalent chronic disease in Fresno County. Its prevalence was constant between 2017 and 2021. Distinct racial and ethnic disparities are evident, with multiple-race non-Hispanic and Black non-Hispanic populations consistently exhibiting higher rates compared to other groups from 2019 to 2022. Similar to hypercholesterolemia rates, while the Asian population generally displays lower HBP rates, a significant rise was observed in 2022. 

U.S. National Data: Nearly half of U.S. adults (47.7%) had hypertension from August 2021 to August 2023. The prevalence is higher in men (50.8%) than in women (44.6%) and increases with age [19.20]. A significant concern is that only about one-fifth (20.7%) of adults with hypertension have their blood pressure adequately controlled. Nationally, high blood pressure is more common in non-Hispanic Black adults (56%) compared to non-Hispanic White (48%), non-Hispanic Asian (46%), or Hispanic adults (39%) [19,20]. 

The consistent prevalence of high blood pressure in Fresno, coupled with low control rates nationally, indicates a challenge in disease prevention and effective management within the county. The disproportionately high rates among Black and multiple-race non-Hispanic populations in Fresno are consistent with the hypothesis that systemic inequities contribute significantly to the groups’ chronic disease burden. The recent increase in HBP prevalence among Asian populations in Fresno in 2022 also aligns with the observed obesity trend in this demographic [21], pointing to potential broader health transitions that require focused attention. A sudden increase in HBP and cholesterol risk among Asian populations may not reflect a true increase in incidence; rather, historically poor screening practices may have caused a delayed spike in diagnostic rates, suggesting underreported true disease prevalence. It is crucial to improve healthcare engagement to accurately report disease prevalence, as it will likely continue to rise as healthcare engagement improves.

Arthritis

Fresno County: According to CDC PLACES 2022 [10], the estimated prevalence of arthritis among adults aged 18 years and older in Fresno was 22.2% (95% CI: 21.4%-23.0%). This compares to 21.0% in California and 26.3% in the U.S. Arthritis has consistently been a predominant chronic condition in Fresno County, with its prevalence rates showing minimal variation between 2018 and 2021 [7]. The average prevalence rate for arthritis in Fresno County for this period is greater than that of the San Joaquin Valley and California. 

U.S. National Data: In 2022, the age-adjusted prevalence of diagnosed arthritis in U.S. adults was 18.9%. Women (21.5%) were more likely to have arthritis than men (16.1%). The prevalence of arthritis correlated with age, escalating from 3.6% in adults aged 18-34 to 53.9% in those aged 75 and older [22]. Furthermore, arthritis prevalence decreased with increasing family income and education levels [23]. Higher rates were reported in non-metropolitan areas.

The consistently high prevalence of arthritis in Fresno aligns with national trends that link arthritis to lower income, less education, and rural living [24]. This observation suggests that the burden of arthritis in Fresno is not solely a reflection of an aging population but is exacerbated by socioeconomic and environmental factors. Fresno's status as a major agricultural city further complicates the situation; however, interventions and policies targeting low-income and less-educated families can mitigate the increased physical wear and tear leading to arthritis.

Diagnosed Diabetes

Fresno County: According to CDC PLACES 2022 [10], the estimated prevalence of diabetes among adults aged 18 years and older in Fresno was 13.1% (95% CI: 11.5%-14.8%). This compares to 11.5% in both California and the U.S. Diagnosed diabetes is a prevalent chronic disease in Fresno County. Prevalence rates among White non-Hispanic and Hispanic groups have fluctuated but remained comparatively low from 2018 to 2022 [7]. The Black non-Hispanic population experienced an upward trend from 2018 to 2020; however, data for 2021 and 2022 were suppressed due to small sample sizes. Conversely, the Asian population's rates initially increased between 2018 and 2019 but showed a marked decrease in 2021 and 2022. Most critically, Fresno County's diabetes death rate was 52% higher than the statewide average and 43% higher than the national average, ranking 56th out of 58 counties in California [25]. 

U.S. National Data: The prevalence of diagnosed diabetes in U.S. adults was 11.3% during August 2021-August 2023 [26]. The total diabetes prevalence (including both diagnosed and undiagnosed cases) was 15.8%. Prevalence is higher in men (12.9% diagnosed) than in women (9.7% diagnosed). It also increases with age and weight status and decreases with educational attainment. Diagnosed diabetes prevalence is highest among American Indian and Alaska Native adults (13.6%), followed by non-Hispanic Black adults (12.1%), adults of Hispanic origin (11.7%), non-Hispanic Asian adults (9.1%), and non-Hispanic White adults (6.9%) [27].

Fresno's exceptionally high diabetes mortality rate, at 52% above the state average, suggests significant challenges in the public health infrastructure for both prevention and disease management. The disease's strong associations with poor diet and physical inactivity [28], paired with the local physician shortage, are community-level factors that should be targeted by proper public health initiatives. Additionally, the elevated mortality rate suggests potential gaps in the care continuum from diagnosis to long-term disease management among Fresno residents.

Asthma

Fresno County: According to CDC PLACES 2022 [10], the estimated prevalence of current asthma among adults aged 18 years and older in Fresno was 10.9 (95% CI: 9.5%-12.1%). This compares to 8.8% in California and 10.3% in the U.S. Asthma is one of the most prevalent chronic diseases in Fresno County [7]. A 2022 survey in South Fresno found that 12% of residents had been diagnosed with asthma [29].

U.S. National Data: Current asthma prevalence among U.S. adults was 8.9% in 2023 [30]. It is more common among lower socioeconomic groups, with a prevalence of 10.4% for those living below 100% of the federal poverty threshold. Racial and ethnic disparities show higher rates in Black non-Hispanic (10.9%), American Indian/Alaska Native non-Hispanic (12.3%), and multiple-race Non-Hispanic (10.3%) populations compared to White non-Hispanic (7.6%), Asian non-Hispanic (4.1%), and Hispanic (6.4%) adults.

Fresno's elevated asthma prevalence is strongly correlated with its poor air pollution, which consistently ranks among the worst in the nation [31]. Fresno, surrounded by mountains and adjacent to extensive agricultural land, is located within a geographic basin where dust and airborne pollutants accumulate. Additionally, Fresno lies in a topographically depressed valley, which restricts air circulation and traps pollutants near the ground, exacerbating long-term exposure to harmful particulate matter. Given Fresno's persistent ranking among the worst U.S. cities for air quality, it is likely that environmental remediation is essential to significantly reduce asthma prevalence. Of less significance, pervasive poverty in Fresno may further increase asthma prevalence, as part of a nationally established inverse relationship between socioeconomic status and asthma prevalence [32].

Coronary Heart Disease

Fresno County: According to CDC PLACES 2022 [10], the estimated prevalence of coronary heart disease (CHD) among adults aged 18 and older in Fresno was 6.3 (95% CI: 5.6%-7.0%). This compares to 5.9% in California and 6.8% in the U.S. CHD is a prevalent chronic disease in Fresno County. Heart attack mortality rates in Fresno County (2018-2021) are consistently higher than both regional and statewide averages [7]. The mortality rate for the Black population shows the highest figures, peaking around 2020 before a slight decline. White non-Hispanic and multi-race non-Hispanic populations maintain relatively stable and lower rates.

U.S. National Data: Heart disease is the leading cause of death for men, women, and most racial and ethnic groups in the U.S. Approximately 1 in 20 U.S. adults aged 20 and older (~5%) have CHD, which was the leading cause of death for 371,506 people in 2022. Key risk factors for CHD include diabetes, being overweight or obese, an unhealthy diet, physical inactivity, and excessive alcohol use [33].

The elevated CHD prevalence in Fresno is a direct consequence of the risk factors mentioned, which, of significant note, are largely preventable [34]. The county's high rates of obesity (38.6%), diagnosed diabetes (13.1%), and hypertension (30.8%) predispose its population to an elevated risk for adverse cardiovascular events [7]. Higher heart attack mortality, especially among the Black population, reflects the compounded impact of uncontrolled, underlying metabolic conditions. This pattern may be attributed to limited access to preventive care and cholesterol screening, neighborhood-level stressors, and chronic exposure to environmental and nutritional inequities that negatively impact these underserved populations.

Stroke

Fresno County: According to CDC PLACES 2022 [10], the estimated prevalence of stroke among adults aged 18 years and older in Fresno was 3.6 (95% CI: 3.3%-3.9%). This compares to 2.2% in California and 3.6% in the U.S. Although stroke is an acute medical event, its long-term complications, including cognitive, mobility, and speech difficulties, justify it as a prevalent chronic disease in Fresno County [7].

U.S. National Data: Stroke is the fifth leading cause of death in the U.S. and a significant cause of severe long-term disability. U.S. self-reported stroke prevalence increased by 7.8% from 2.7% (2011-2013) to 2.9% (2020-2022) [35]. The risk of experiencing a first stroke is nearly twice as high for non-Hispanic Black adults compared to White adults. Stroke prevalence is also higher among non-Hispanic American Indian or Alaska Native, non-Hispanic Native Hawaiian or Pacific Islander, and Black adults than among White adults. Furthermore, it is more prevalent among adults with less than a college degree. Key risk factors for stroke include high blood pressure, high cholesterol, smoking, obesity, and diabetes.

The high prevalence of stroke in Fresno (3.6%) can be attributed to uncontrolled risk factors endemic to the county. With nearly a third of the adult population hypertensive (30.8%) and over one in ten diagnosed with diabetes (13.1%), the neurological and vascular systems are placed under sustained pressure, thus elevating the stroke rate to an anticipated yet alarmingly poor health outcome [7, 36]. The racial disparities in stroke risk and prevalence observed in Fresno's diverse, disadvantaged communities are likely mirrored and potentially amplified compared to national levels. The burden of stroke in Fresno appears to be strongly correlated with widespread prevalence and poor management of its major risk factors. Thus, community involvement is crucial in addressing these issues.

Underlying causes and risk factors for poor outcomes in Fresno County

The underlying causes and risk factors contributing to health disparities in Fresno County are deeply interconnected, each playing a critical role in shaping community well-being. These factors encompass socioeconomic status (including poverty, income, and education), access to healthcare (encompassing insurance, provider shortages, and cost barriers), environmental and occupational exposures (such as air quality and pesticides), lifestyle factors (including diet, physical inactivity, and smoking), and education and language barriers (covering health literacy and linguistic isolation). These elements form a complex web that influences health outcomes and necessitates comprehensive solutions to address each contributing factor.

**Figure 2:**
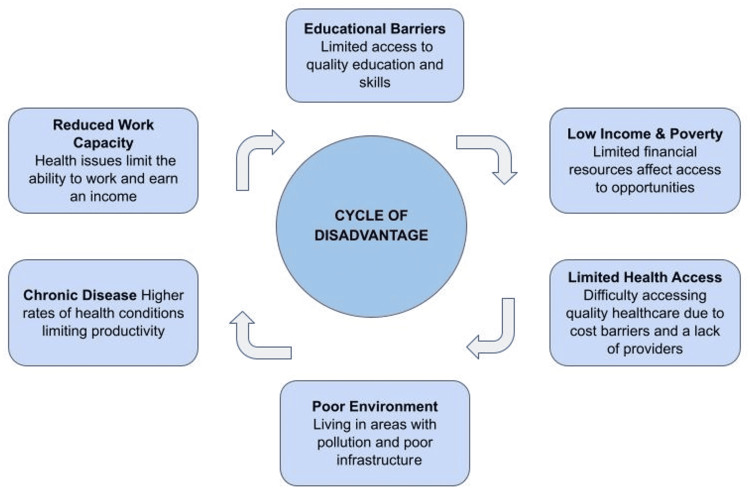
Cycle of Disadvantage Delineation of a reinforcing cycle of disadvantages where socioeconomic and health-related factors are reciprocally linked. The cycle demonstrates that low income and poverty restrict access to quality healthcare. This limitation, often exacerbated by poor environmental conditions such as pollution and inadequate infrastructure, contributes to a higher incidence of chronic disease. Image created by the authors using Google Slides (Alphabet Inc, Mountain View, USA).

Chronic disease impairs work capacity, diminishing an individual's ability to maintain stable employment and earn income. This is compounded by educational barriers that limit access to skills and qualifications. Together, these factors reduce access to quality healthcare, furthering the existing health disparities in these disadvantaged groups. The resulting reduction in earnings reinforces the initial state of poverty, perpetuating a cycle of poor health and economic hardship that can span generations. This interdependence creates a self-reinforcing cycle, as shown in Figure 2, where each disadvantage compounds the following. This underscores the importance of utilizing collaborative, multi-sectoral solutions over isolated interventions.

Poverty & Education Disparities

Poverty and Income: Fresno County faces a significantly higher rate of child poverty, recorded at 36.5%, in sharp contrast to California's overall rate of 20.8% [8]. Furthermore, specific racial and ethnic groups within Fresno County are disproportionately affected by poverty; for instance, African Americans (39%), American Indians and Alaska Natives (33%), and Hispanic/Latino populations (32%) experience significantly higher poverty rates compared to their White counterparts (12%). Overall, 45% of residents in Fresno and surrounding Central Valley counties have incomes below 200% of the federal poverty level, a higher proportion than the 30% observed statewide [37]. This extreme poverty rate acts as a fundamental driver of widespread health disparities.

Education: Educational attainment levels in the Central Valley are a concern, with three metropolitan areas (Visalia, Merced, and Bakersfield) ranking among the 10 U.S. metropolitan areas with the lowest average education in a 2013 report [5]. According to the 2022 Programme for the International Assessment of Adult Competencies (PIAAC)survey, approximately 28% of California adults struggle with basic reading, and in the Central Valley, especially among immigrant populations, over 40% have difficulty with simple English literacy. Lower educational attainment is consistently linked to a higher prevalence of chronic diseases such as obesity, diabetes, and stroke [13]. 

The pervasive poverty in Fresno County, particularly its disproportionate impact on underserved groups, creates a vicious cycle. In this, limited finances restrict access to healthy food, safe environments for physical activity, quality education, and adequate healthcare, fueling the chronic disease epidemic. This pattern underscores that health interventions cannot be solely clinical; they must integrate broader socio-economic upliftment strategies. The excessive impact on minority groups points to systemic inequities that necessitate targeted and culturally sensitive multi-sectoral interventions. Policymakers play a crucial role in implementing these primary health strategies by addressing economic opportunities and educational attainment.

Limited Healthcare Access & Affordability

Insurance Coverage: In 2021, 7.00% of California's residents lacked health insurance coverage. While this number signifies overall improvement from the 18.54% uninsured rate in 2016, Central Valley, including Fresno, continues to face significant challenges in healthcare access [2]. Between 2016 and 2020, 39.9% of Fresno County residents were enrolled in Medi-Cal, compared to 24.2% statewide, highlighting the region's higher dependence on public insurance [38].

Provider Shortages: The San Joaquin Valley, where Fresno is located, averages fewer primary care doctors (48 per 100,000 residents) and specialists (80 per 100,000) compared to recommended levels and urban regions of California [5]. More than half (56%) of Central Valley residents perceive a shortage of mental health providers in their communities. Thus, these patients may seek care outside the Valley due to the subsequent unavailability or extended waiting times of local services.

Cost Barriers and Medical Debt: Nearly half (48%) of Central Valley residents carry medical debt, a greater proportion than the 36% observed in California overall [38]. A striking 63% of adults in the Fresno area reported skipping or delaying healthcare, such as foregoing doctor visits or not following through on recommended tests or treatments, due to financial concerns in 2024.

Timeliness and Regular Care: One-half (50%) of Central Valley residents reported waiting longer than they considered reasonable for a physical health appointment [38]. Additionally, Central Valley residents are less likely (78% vs. 85% elsewhere in California) to have a regular doctor or healthcare provider.

Provider shortages, high medical debt, and cost-related care compound to create a critical gap in the healthcare safety net in Fresno. When residents delay or forgo care due to financial constraints or lack of access, chronic conditions like diabetes, hypertension, and heart disease remain poorly managed. Uncontrolled conditions give rise to exacerbations, complications, and ultimately higher rates of hospitalization and mortality, intensifying the burden on hospitals and emergency services. This increase in healthcare costs, in addition to an unfavorable payer mix of a high Medi-Cal-to-commercial-insurance proportion, strains the local “safety net” hospital systems and their already limited resources. This feedback loop, where limited healthcare access drives poor health outcomes, then impairs the health systems' capacity to provide quality care.

Environmental Health & Safety Concerns

Air Quality: Fresno County is characterized by some of the nation's most significant environmental inequalities. In 2022, Fresno ranked highest for short-term particle pollution and second highest for year-round particle pollution [29]. High concentrations of particle pollution are scientifically linked to increased hospitalizations, asthma attacks, cardiovascular disease, lung cancer, chronic obstructive pulmonary disease (COPD), heart attacks, and premature death [39]. Most South Fresno residents (61%) report experiencing trouble resting due to air pollution. Specific pollutants like carbon monoxide (CO) and fine particulate matter (PM2.5) are identified as contributors to heart disease and chronic respiratory disease, respectively.

Pesticide Exposure: The Central Valley, including Fresno, is a major agricultural region characterized by extensive pesticide use, with 181 million pounds of active ingredients applied in California in 2022 [40]. Farmworkers, a predominantly Latino immigrant population in Fresno County, face elevated levels of exposure to hazardous pesticides [41, 42]. Chronic exposure to certain pesticides has been linked to various cancers, increased rates of Parkinson's disease, kidney disease (including end-stage renal disease), and respiratory issues such as asthma and wheezing [40, 43]. These detrimental health effects are particularly pronounced in children due to their unique developmental, dietary, and physiological factors. A significant concern is that many pesticide poisonings go unreported, often due to farm workers' fear of job loss or inability to afford medical care [44].

The dual burden of severe air pollution and widespread pesticide exposure in Fresno creates a toxic environment that directly contributes to the high prevalence of respiratory, cardiovascular, and neurological chronic diseases in Fresno. Agriculture and transportation, which are key industries that drive Fresno's economy, are increasingly generating pollutants that cause and worsen chronic diseases, creating an inherent conflict between economic prosperity and public health. This largely affects low-income and minority communities, particularly farm workers, highlighting a profound environmental injustice where economic activity comes at a direct cost to public health. Farmworkers are often vulnerable people due to limited access to legal protection and low socioeconomic status. Because of this, they can hesitate to report exposures, suggesting that the actual burden of these exposures is likely underestimated [45].

Unhealthy Lifestyles & Food Insecurity

Diet and Food Insecurity: Despite its status as the "heart of California's Central Valley" and a leading agricultural producer, Fresno County residents struggle with food insecurity and limited access to fresh fruits and vegetables [46]. Economic disparities and logistical barriers contribute to creating "food deserts" within the county, where healthy food options are either scarce or unaffordable [47]. Fresno consistently ranks among the top five metropolitan areas nationally for food hardship [48]. Approximately 13.6% of Fresno County residents experienced food insecurity in 2021, with higher rates observed among Black (24%) and Hispanic (17%) residents compared to White (8%) residents.

Physical Inactivity: Inadequate physical activity is associated with an estimated $117 billion in annual healthcare costs and significantly increases the risk of developing chronic diseases [49]. Nationally, only one in four U.S. adults fully meets recommended physical activity guidelines. While California adults generally exhibit higher rates of meeting physical activity recommendations (70.5% in 2017), Fresno County faces distinct challenges. Poor investment in parks, bike lanes, and walking paths, particularly in the underserved southeast and southwest areas, severely limits physical activity opportunities [50]. In 2011-2012, only 22.5% of children aged 5-17 in Fresno County engaged in regular physical activity [51].

Smoking: Smoking is a major modifiable risk factor for numerous chronic diseases, contributing to over 480,000 deaths annually in the U.S. and increasing the risk of cancer, heart disease, stroke, lung diseases, and diabetes [52]. The smoking rate in the city of Fresno was 14.5% in 2022, but some surrounding rural areas recorded significantly higher rates, such as Orange Cove (22.0%) and Mendota (21.4%) [53, 54].

The paradox of food insecurity in an agricultural hub, coupled with limited infrastructure for physical activity and elevated smoking rates in specific areas, creates a confluence of factors conducive to lifestyle-driven chronic diseases. These factors are not merely individual choices but are profoundly shaped by systemic socioeconomic disparities and urban design, making healthy living a privilege rather than an accessible norm. The "food desert" phenomenon in an agricultural heartland is a contradiction that limits access to healthy food for the people who produce it. Similarly, the absence of safe and accessible spaces for physical activity, combined with high smoking rates in vulnerable communities, suggests that healthy lifestyle choices are structurally impeded. Compounding this, poor air quality in the Central Valley frequently makes outdoor physical activity unsafe, especially for individuals with asthma, COPD, or other chronic health conditions.

Limited Health Literacy & Deficiency in Culturally Competent Care

Linguistic Isolation: Linguistic isolation is defined as a household where no one over 14 speaks English proficiently. Specific zip codes within Fresno County exhibit linguistic isolation rates that are more than double the California average and three times the national average [55]. This communication barrier limits accessibility to healthcare, public benefits, and community resources [56]. Vital information on prevention, treatment options, and service eligibility from these sources is often available exclusively in English. As a result, linguistically isolated residents face delays in care and reduced utilization of preventive services and thus have poorer health outcomes. Patients may avoid discussing sensitive health concerns through interpreters, who are often their children. Coordinating appointments around an interpreter's availability further impedes timely access to care.

Impact on Care: Language barriers and low health literacy create a significant communication gap between the healthcare system and non-English speakers. These factors directly impede effective chronic disease management, as patients may not fully comprehend their diagnoses, treatment plans, or the critical importance of adherence, leading to poorer outcomes and increased healthcare utilization for preventable complications [55, 57].

This highlights the need for culturally and linguistically appropriate health education, widespread deployment of community health workers (CHWs) who can bridge these gaps, and systemic changes within healthcare organizations to prioritize organizational health literacy [52, 53].

Inadequate Rural Infrastructure & Transportation

Geographic and Transportation Challenges: Fresno County spans nearly 6,000 square miles, encompassing vast, sparsely populated rural areas [50]. This expansive geography presents significant challenges for providing high-quality public transit services. Nearly 80% of Fresno County's transit riders are entirely dependent on public transportation, a figure that doubles the national average, and these individuals often work in low-wage jobs. Moreover, 20% of individuals share a vehicle or do not have access to a car [50]. Existing public transit routes, such as the southeast route connecting Kingsburg to Fresno, do not offer frequent service, further limiting accessibility.

Transportation is an often-overlooked social determinant of health. Inaccessibility to reliable transportation is a significant barrier to healthcare access, causing rescheduled or missed appointments, delayed care, and missed or deferred medication use. These consequences directly contribute to poorer management of chronic illnesses and worse health outcomes, particularly for individuals with lower incomes or who are uninsured. Rural communities situated along major transportation corridors are often socioeconomically disadvantaged and burdened by pollution [50]. Limited transportation options hinder their access to essential services such as employment, healthcare, and other necessary amenities.

Public health implications and recommendations

Significance of Regional Disparities

The presented data demonstrate that Fresno County bears a disproportionate burden of chronic diseases compared to both the SJV and California state averages, despite California's overall strong health performance [8]. The profound inequities in health outcomes and access to resources largely affect a large geographic region within a normally high-performing state. The concentration of chronic diseases in Fresno County is inextricably linked to a complex interplay of socioeconomic disadvantage, environmental hazards, and systemic barriers to healthcare access and healthy lifestyle options. These factors create a self-perpetuating cycle of poor health that is difficult to disrupt without targeted, multi-faceted interventions.

Furthermore, these disparities manifest along racial and ethnic lines, with Black, Hispanic, and American Indian/Alaska Native populations frequently experiencing higher prevalence rates for obesity, high blood pressure, diabetes, and stroke, alongside greater levels of poverty [7]. Fresno's health crisis is a strong indicator of the inherent limitations in statewide health policies that do not adequately account for localized, compounded social and environmental determinants of health. The persistence and severity of Fresno's health disparities, even within a high-performing state, serve as a warning that "average" state-level health successes can obscure profound local crises and internal inequities, underscoring that targeted regional investment is an ethical and public health imperative [8]. Addressing these disparities requires restructuring health resource allocation, shifting toward equity-driven public health policy, and prioritizing high-need regions.

This case study of Fresno County serves as a critical dialogue between clinical medicine, public health policy, urban planning, and environmental science, demonstrating that siloed approaches are insufficient.

Structural Interventions to Address Socioeconomic Determinants of Health

**Figure 3:**
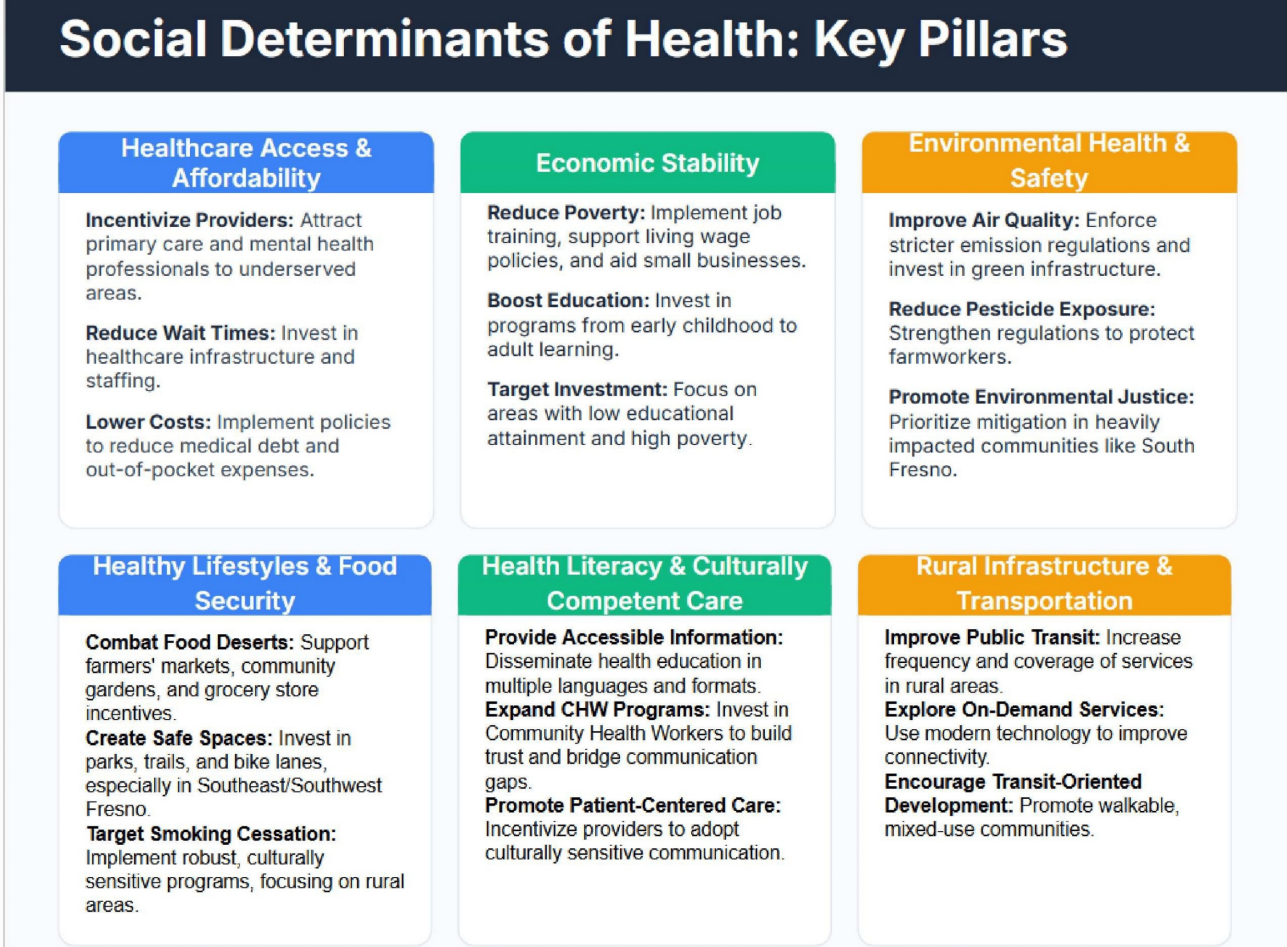
A call for structural change in Fresno County A proposed multi-sectoral framework for structural intervention to address chronic disease disparities in Fresno County. Image created by the authors using Google Slides (Alphabet Inc., Mountain View, USA).

Recognizing that chronic disease burden often stems from socioeconomic inequities, sustainable improvements in health outcomes depend on addressing these foundational drivers. As illustrated in Figure 3, the following six root causes warrant targeted attention to reduce disparities and promote long-term community health.

Enhancing Healthcare Access and Affordability

Expand Primary Care and Specialty Providers: Implement incentives, such as loan forgiveness programs or rural practice subsidies, to attract and retain healthcare professionals, particularly primary care physicians and mental health providers, in underserved areas of Fresno County. Invest in healthcare infrastructure and staffing to significantly reduce appointment wait times and ensure timely access to physical and mental health services [58].

Address Cost Barriers: Develop and implement policies to reduce out-of-pocket costs and medical debt for residents. This could involve expanding Medi-Cal enrollment assistance, providing targeted subsidies, or establishing community-based financial counseling services [58].

Reducing Poverty and Improving Educational Attainment

Poverty Reduction Initiatives: Implement comprehensive programs to increase economic stability and reduce poverty, focusing on disproportionately affected racial and ethnic groups. Such initiatives include job training programs, advocating for living wage policies, and supporting small businesses in disadvantaged communities [6,53].

Educational Attainment: Invest significantly in educational programs spanning from early childhood through adult learning, targeting areas with historically low average education levels. This is crucial because education consistently correlates with improved health outcomes [5, 17].

Chronic disease burden is not merely a medical problem but also a pervasive societal issue. Investing in education and economic opportunities is a long-term public health strategy. It acknowledges that health is fundamentally shaped by where people live, learn, work, and play, rather than solely within clinical settings.

Improving Environmental Health and Safety

Air Quality Improvement: Implement and rigorously enforce stricter industrial emissions and vehicle pollution regulations. Invest in green infrastructure and expand public transit options to reduce reliance on private vehicles, which are major contributors to air pollution. Prioritize intensified air quality monitoring and mitigation efforts in heavily impacted communities, particularly in south Fresno [5, 17].

Pesticide Exposure Mitigation: Strengthen regulations and enforcement mechanisms to reduce farmworker exposure to hazardous pesticides. Support new or ongoing research into safer agricultural practices and provide accessible, comprehensive services for farm workers, including education on pesticide hazards and confidential reporting mechanisms that protect workers from retaliation [35, 38].

Environmental health is foundational to public health in Fresno. The county relies heavily on agriculture, and the transportation economic structure inherently generates ecological health risks. Therefore, policy must balance economic development and public health protection. A “health in all policies" approach that integrates health considerations into land use, transportation, and agricultural planning. Furthermore, addressing the power dynamics that allow vulnerable populations to be disproportionately exposed to environmental hazards is essential for achieving true ecological justice.

Promoting Healthy Lifestyles and Food Security

Combat Food Deserts: Implement policies and programs to increase access to affordable, nutritious food in underserved areas. This could involve supporting local farmers' markets, establishing community gardens, and offering incentives for grocery stores to establish food deserts. Expand existing programs like Project Food Box and Food to Share that deliver fresh produce directly to food-insecure families [40, 41].

Increase Physical Activity Opportunities: Invest in developing safe and accessible infrastructure for walking and biking, including parks, trails, and dedicated bike lanes, particularly in the underserved communities of southeast and southwest Fresno. Promote community-based physical activity programs that are culturally relevant and accessible to all residents [50].

Targeted Smoking Cessation: Implement robust, culturally sensitive tobacco cessation programs, with a specific focus on rural areas that exhibit high smoking rates. Strengthening current public health campaigns aimed at reducing smoking [50, 51].

The surrounding environment heavily influences individual lifestyle choices. If healthy food options or safe spaces for physical activity are unavailable or unaffordable, promoting "healthy choices" is insufficient and can inadvertently place blame on individuals. Therefore, policy must focus on creating environments where healthy choices become easy choices and address the market failures and historical underinvestment in infrastructure within disadvantaged communities.

Strengthening Health Literacy and Culturally Competent Care

Culturally and Linguistically Appropriate Health Education: Develop and disseminate health information in multiple languages and formats that are accessible and understandable to diverse populations [55].

Expand Community Health Worker (CHW) Programs: Investing in and professionalizing CHW programs is crucial in bridging communication gaps, building trust, and connecting residents with essential resources for chronic disease management. To maximize impact, implement strategies to rebuild trust between communities and healthcare providers, acknowledging historical grievances and actively engaging communities in designing and implementing health interventions [55].

True health literacy extends beyond translation; it involves making health information and services usable and understandable within a patient's cultural context. Often rooted in historical experiences, mistrust is a significant barrier to care. CHWs are vital because they are trusted community members who can navigate cultural nuances and build rapport. Policy must support the integration of CHWs into the broader healthcare system and incentivize healthcare providers to adopt patient-centered approaches. Recognizing that effective communication is a critical aspect of improving clinical outcomes.

Investing in Rural Infrastructure and Transportation

Improve Public Transit: Increase the frequency and coverage of public transit services in rural areas of Fresno County. This includes exploring on-demand and app-based services to improve connectivity to healthcare facilities, grocery stores, and employment centers [59].

Support Transit-Oriented Development (TOD): Encourage land use planning that promotes mixed-use developments around transit corridors. This promotes walkable and bikeable communities that reduce reliance on private vehicles and encourage physical activity [59].

Even if healthcare services exist, geographic isolation and poor transit infrastructure prevent access to a large portion of the population, particularly the transit-dependent and residents of rural areas [60]. These barriers contribute to delayed diagnoses, suboptimal chronic disease management, and increased reliance on emergency services [61]. Investment in rural transit is a long-term strategy that also acts as an investment in chronic disease prevention and management.

To address these compounding disparities, we propose a coordinated, multi-sectoral framework for structural intervention targeting the root causes of chronic disease in Fresno County (Figure 3).

## Conclusions

The data presented in this review leave little ambiguity: adult obesity is 38.6%. Diabetes mortality is 52% above the statewide average. Stroke prevalence exceeds California's rate by more than 60%. Asthma is disproportionately elevated in communities facing the nation's worst air quality. This chronic disease burden of Fresno County is not the product of individual failure but of accumulated structural disadvantage. Fresno's health crisis reflects the cumulative weight of poverty, environmental injustice, provider shortages, and systemic barriers to care, all concentrated within one of California's most economically productive regions. The fact that this crisis persists within a high-performing state underscores a critical lesson: statewide averages can conceal profound local emergencies, and policies designed around the mean will consistently fail communities at the margins. The evidence therefore necessitates a paradigm shift away from isolated clinical interventions toward a holistic public health framework that targets the structural roots of disease.

This paper proposes an integrated, multi-sectoral strategy organized around six interdependent pillars: enhancing healthcare access and affordability; reducing poverty and improving educational attainment; improving environmental health and safety; promoting healthy lifestyles and food security; strengthening health literacy and culturally competent care; and investing in rural infrastructure and transportation. These pillars do not function independently: physician shortages compound uncontrolled hypertension, food deserts accelerate diabetes progression, linguistic isolation delays diagnosis, and inadequate transit prevents follow-up care, forming the self-reinforcing cycle of disadvantage this paper has documented. Implemented with fidelity and guided by community stakeholders and local public health expertise, this framework holds measurable potential: reductions in preventable hospitalizations, narrowing of racial and ethnic mortality gaps, and decreased chronic disease incidence across Fresno's most vulnerable populations. Beyond the county itself, Fresno's structural conditions mirror those of communities throughout the Central Valley and across the nation, where agricultural productivity coexists with entrenched socioeconomic deprivation, positioning this framework as a replicable blueprint for health justice in similarly affected regions.
